# Prize Essays for 1837-8

**Published:** 1837-01

**Authors:** 


### PRIZE ESSAYS FOR 1837-8.

### 1. *By the Royal Academy of Medicine of Paris.*

1. The Academy offers a prize of two thousand francs for the best Essay on "the Physiological History of Menstruation, the influence exercised by this function on diseases, and the effect of diseases upon the secretion." To be awarded in 1837.

2. *Portal Prize.* On "the History of the Discoveries relating to the Venous System, from Morgagni until the present day, and on the influence which these discoveries have had upon the knowledge and treatment of diseases." Prize, six hundred francs, to be awarded in 1838.

3. *Bernard Prize.* On "the Influence of Physical and Moral Education in producing Excitability *(surexcitation)* of the Nervous System, and on the Diseases resulting from such Excitability." The prize, of fifteen hundred francs, will be awarded in 1838.

N.B. The essays, enclosed in the usual form, are to be sent to the secretary's office at the Academy, before the 1st of March, 1837 and 1838.

### 2. *By the Society of Corresponding Physicians at St. Petersburg.*

#### ON HOMŒOPATHY.

The Society of Corresponding Physicians at St. Petersburg, acting on the conviction that all the cases of disease under the homœopathic treatment are only examples of the natural progress of morbid conditions of the organism, such as rational physicians should seldom be permitted to produce by their intentionally doing nothing, requires—That all the recorded cases in the whole range of homœopathic literature be classed, critically examined, and compared one with the other, so that the progress of development of entire classes and genera of disease, as well as individual cases, may be placed in as clear a point of view as possible; that the results of these enquiries be compared with those observed under the Hippocratic method of treatment; and also that the symptoms which usually precede the favorable and unfavorable termination of diseases homœopathically treated be described, as well as the change of form or metastasis of morbid affections, when these occur. The Society wishes, by means of this task, to give rise to a complete and critical application of the homœopathic cases of disease already published, for the purpose of discovering the laws of development of the pathological phenomena in the human organism, so that the remarkable schism of the Hahnemannians in rational medicine may not be without profit to the latter. All recriminations against homœopathy as a science, and against its supporters as physicians, must be avoided. The essays are to be written in the Russian, Latin, French, or German language; they must be sent in, with motto and name under seal, free of expense, by the

15th (27th) July, 1837, addressed to Mr. State-Counsellor Fuss, permanent secretary of the Imperial Academy of Science at St. Petersburg.

The prize, fifty Dutch ducats, (about 24*l.*) will be awarded on the anniversary of the Society, 26th November, (8th December,) 1837, and the accepted essay afterwards published by the Society; for which the author shall receive the usual remuneration, over and above the prize.

*St. Petersburg; Jan.* 28, 1836. Dr. Weisse,

Dr. Seidlitz.

*Med. Zeitung.* 30 *März,* 1836.

3. *By the St. Petersburg German Medical Society.*

ON THE EGYPTIAN OPHTHALMIA.

A gentleman, having suffered from the destructive influence of the Egyptian ophthalmia, and desirous, for the benefit of society, that the nature of the disease, and most effectual means of curing it, should be discovered and promulgated, has presented to the German Medical Society at St. Petersburg the sum of one thousand rubles in money, (about 200*l.* sterling,) upon eondition that the Society propose a prize essay on the above disease, examine the dissertations of candidates, and reward the author of the paper which, in their judgment, shall be the most satisfactory, with the full amount of the money thus intrusted to them. The Society, joyfully assenting to the wishes of the donor of this prize, hereby invite all practitioners in diseases of the eye to send in a complete Treatise on the Egyptian Ophthalmia.

The treatises must be written either in Latin, French, English, or German; must be sent in by the 15th (27th) September, 1837, with the motto and the name of the author sealed up, free of expense, and directed to the secretary of the German Medical Society at St. Petersburg. The presentation of the prize will take place on the foundation day of the Society, January 21, (February 2d,) 1838. The successful essay will be published in the next ensuing volume of the accepted miscellaneous treatises of the Society, with honorable distinction: the others will be returned to the respective authors, (whose names will remain unknown,) if required.

*St. Petersburg; Jan.* 21, 1836. Dr. Busch, Director.
Dr. Seidlitz, Secretary.

*Medicinische Zeitung.* 30 *März,* 1836.

---

HOMŒOPATHY.

